# Characterization of exosomal release in bovine endometrial intercaruncular stromal cells

**DOI:** 10.1186/s12958-016-0207-4

**Published:** 2016-11-09

**Authors:** Yong Qin Koh, Hassendrini N. Peiris, Kanchan Vaswani, Sarah Reed, Gregory E. Rice, Carlos Salomon, Murray D. Mitchell

**Affiliations:** University of Queensland Centre for Clinical Research, The University of Queensland, Brisbane, Queensland Australia

**Keywords:** Bovine, Intercaruncular, Hypoxia, Exosomes

## Abstract

**Background:**

Cell-to-cell communication between the blastocyst and endometrium is critical for implantation. In recent years, evidence has emerged from studies in humans and several other animal species that exosomes are secreted from the endometrium and trophoblast cells and may play an important role in cell-to-cell communication maternal-fetal interface during early pregnancy. Exosomes are stable extracellular lipid bilayer vesicles that encapsulate proteins, miRNAs, and mRNAs, with the ability to deliver their cargo to near and distant sites, altering cellular function(s). Furthermore, the exosomal cargo can be altered in response to environmental cues (e.g. hypoxia). The current study aims to develop an in vitro system to evaluate maternal-embryo interactions via exosomes (and exosomal cargo) produced by bovine endometrial stromal cells (ICAR) using hypoxia as a known stimulus associated with the release of exosomes and alterations to biological responses (e.g. cell proliferation).

**Methods:**

ICAR cells cultured under 8 % O_2_ or 1 % O_2_ for 48 h and changes in cell function (i.e. migration, proliferation and apoptosis) were evaluated. Exosome release was determined following the isolation (via differential centrifugation) and characterization of exosomes from ICAR cell-conditioned media. Exosomal proteomic content was evaluated by mass spectrometry.

**Results:**

Under hypoxic conditions (i.e. 1 % O_2_), ICAR cell migration and proliferation was decreased (~20 and ~32 %, respectively) and apoptotic protein caspase-3 activation was increased (∼1.6 fold). Hypoxia increased exosome number by ~3.6 fold compared with culture at 8 % O_2_. Mass spectrometry analysis identified 128 proteins unique to exosomes of ICAR cultured at 1 % O_2_ compared with only 46 proteins unique to those of ICAR cultured at 8 % O_2_. Differential production of proteins associated with specific biological processes and molecular functions were identified, most notably ADAM10, pantetheinase and kininogen 2.

**Conclusions:**

In summary, we have shown that a stimulus such as hypoxia can alter both the cellular function and exosome release of ICAR cells. Alterations to exosome release and exosomal content in response to stimuli may play a crucial role in maternal-fetal crosstalk and could also affect placental development.

## Background

In dairy cattle, the average gestation length is approximately 282 days. The placenta is epitheliochorial, cotyledonary and non-deciduate [1]. Placentation is restricted to the aglandular maternal caruncles, where the fetal cotyledons come into contact with each other [2, 3]. They then form the placentome for maternal-fetal exchange of oxygen, nutrients and waste products. The glandular intercaruncular regions are associated with preserving the uterus in a state of quiescence and allowing a progressive uterine hypertrophy to accommodate the increasing needs of the growing feto-placental unit [4]. The uterine glands present in the intercaruncular endometrial areas secrete and release histotroph that is crucial for conceptus survival and growth [5] and is transported into the fetal circulation via the placental areolae. The establishment of a successful pregnancy requires the interactions between the endometrial cells and the early conceptus during maternal recognition of pregnancy [6, 7].

Cells located within intercaruncular region and associated with maternal fetal crosstalk include cells of stromal (intercaruncular stromal cell; ICAR) and epithelial origin. Both cell types are known to produce prostaglandins (e.g. PGF_2α_) and have immunomodulatory functions [8, 9]. Interactions between these cells may also play a pivotal role in endometrial receptivity during early pregnancy as was reported in a co-culture study that human endometrial stromal cells can mediate epithelial cell function by promoting differentiation and inhibiting proliferation of endometrial epithelial cells [10]. In the bovine, endometrial stromal cells (as utilized in the current study) are known to differentially regulate the production of prostaglandins and enzymes related to the production of prostaglandins, in response to specific stimuli (e.g. inflammatory mediators and interferon tau) [8, 11]. ICAR cells were a kind gift from Professor Michel A. Fortier (Université Laval, Québec). ICAR cells are a transformed cell-line derived from the intercaruncular region of the bovine endometrium [12]. ICAR cells can be propagated while still maintaining the phenotypical characteristics of these cells which include the presence of SV40 TAG and the vimentin-positive and cytokeratin-negative features that support the stromal phenotype of these cells [8, 13]. This study aimed to evaluate the effect of a known stimulus of exosome release on the production of exosomes by ICAR cells.

In recent years, evidence has emerged from studies in humans [14] and several other animal species [15–18] that exosomes are secreted from the endometrium and trophoblast cells and may play important roles at the conceptus-endometrial interface during early pregnancy. Exosomes are specific subsets of extracellular vesicles (smaller than 1000 nm) [19] that could provide insights into an alternative new explanation for the crosstalk between cells. Exosomes (30–120 nm) are stable extracellular lipid bilayer vesicles arising from the inward budding of multivesicular bodies and released via an exocytic pathway to the extracellular environment with the capacity to modify the biological function of target cells [20]. Exosomes provide a mechanism of cell-to-cell communication in physiological and pathological conditions and may be influenced by neighboring cells, distant tissues or local environmental factors. There is considerable evidence that hypoxia is a potent stimulant to the release of exosomes [21–24]. It is also a useful investigatory agent since a lower-than-normal oxygen tension *in utero* can influence many developmental events with potentially lifelong consequences [25, 26].

Hypoxia is a well-known stimulus of exosome release as seen in breast cancer cells, endothelial cells and human trophoblasts [24, 27, 28]. Alterations have been documented in both the number of exosomes released as well as differences in the content (cargo) of the exosomes [24, 27, 29]. This study aimed to test the hypothesis that hypoxia as a known stimulus of exosome release (and altered biological response) will modify the phenotype of bovine endometrial stromal cells affecting their migration, proliferation, apoptosis as well as altering both the release and cargo of the exosomes generated.

## Methods

### Aim

This study investigated the effect(s) of a hypoxic environment on the function of bovine endometrial cells. In particular, alterations to migration, proliferation and apoptosis. Moreover, this study evaluated alterations to the release and cargo content of exosomes generated by bovine endometrial cells, when cultured under hypoxia.

### Endometrial cell line

A well characterized bovine endometrial intercaruncular stromal cell line (ICAR cells) was utilized for the current study [8, 30]. ICAR cells were a kind gift from Professor Michel A. Fortier (Université Laval, Québec). ICAR cells were maintained in 175 cm^2^ (T175, Corning Costar) culture flasks supplemented with exosome-free media (1640 Roswell Park Memorial Institute (RPMI) medium (Invitrogen, Life Technologies) with 10 % heat-inactivated fetal bovine serum (Bovogen, Interpath services Pty Ltd) depleted of exosomes by ultracentrifugation (100,000 *g* for 20 h at 4 °C) and 1000 U/mL antibiotic-antimycotic solution (Gibco, Life Technologies) in a humidified cell culture incubator at 37 °C under an atmosphere of 5 % CO_2_-balanced N_2_ to obtain a hypoxic (1 % O_2_) environment or under physiologically relevant conditions (8 % O_2_). Lactate dehydrogenase (LDH) assay was also performed accordingly to the manufacturer’s protocol using the commercially available kit Pierce LDH cytotoxicity assay kit (Thermo scientific) to measure LDH in supernatants of ICAR cells cultured at 8 % O_2_ and 1 % O_2_ and ICAR cell viability was accessed. No significant difference in the LDH activity was observed (data not shown) between 8 % O_2_ and 1 % O_2_, indicating that the viability of ICAR cells was not affected by experimental condition.

### Cell migration assay

The effect of oxygen tension on cell migration was assessed using methods as previously published [31]. Briefly, ICAR cells were plated (30,000 cells per well) and grown to confluence in a 96-well culture plate (Corning Costar) at 1 % O_2_ or 8 % O_2_ oxygen tension and a wound scratch was made on confluent monolayers using a 96-pin WoundMaker (Essen BioScience). Migration assays were performed in the presence of Mitomycin C (100 ng/mL, Sigma–Aldrich) to minimize any confounding effects of cell proliferation. The wound images were automatically acquired every 2 h for 48 h and registered by the IncuCyte software system (Essen BioScience). Data are presented as the Relative Wound Density (RWD, Eizen, v1.0 algorithm). RWD is a representation of the spatial cell density in the wound area relative to the spatial cell density outside of the wound area at every time point (time-curve).

### Cell proliferation assay

Proliferation of ICAR cells was assessed using methods as previously published [28, 31]. In brief, the effect of oxygen tension on ICAR cell proliferation was assessed using a non-labelled cell monolayer confluence approach with a high density phase contrast real-time cell imaging system (IncuCyte™). ICAR cells were seeded at 40,000 cells per well in a 12-well culture plate (Corning Costar) and exposed to oxygen tension at 1 % O_2_ or 8 % O_2_ and the cell confluence (as the proliferation parameter) was measured at 0, 24 and 48 h.

### Cell apoptosis assay

To assess the effect of hypoxia on cell apoptosis, ICAR cells were seeded at 5000 cells per well in 96-well culture plate (Corning Costar) in the presence of CellPlayer Kinetic Caspase-3/7 Apoptosis Assay Reagent (1:5000; Essen Biosciences) and imaged at 48 h with IncuCyte™. Cell apoptosis is determined by the measurement of the number of activated caspase 3/7 fluorescent objects count per mm2 divided by the percentage of cell confluence at 48 h (percentage of the area of field of view covered by cells with the metric ‘phase object confluence’) with the IncuCyte Zoom software using an integrated object counting algorithm.

### Exosome isolation from cell-conditioned media

To study the effect of oxygen tension on exosome release, ICAR cells were incubated at 1 % O_2_ or 8 % O_2_ for 48 h. Exosomes were isolated from ICAR cell culture-conditioned media by successive differential centrifugation steps at 300 × *g* for 10 min and 2000 × *g* for 30 min. The supernatant was filtered through a 0.22-μm filter (Corning Costar) and ultracentrifuged at 100,000 × *g* for 20 h at 4 °C (Sorvall, SureSpin 630/360, Swinging-bucket ultracentrifuge rotor). Another round of ultracentrifugation washing steps was performed at 100,000 × *g* for 2 h at 4 °C (Beckman, Type 70.1 Ti, Fixed angle ultracentrifuge rotor). Exosomes were further enriched by layering on top of a discontinuous iodixanol gradient (OptiPrep, Sigma–Aldrich), which was centrifuged at 100,000 × *g* for 20 h (Beckman, Sw41Ti, Swinging-bucket ultracentrifuge rotor). Twelve fractions were obtained and diluted in 10 mL PBS (Gibco, Life Technologies). The fractions were washed with PBS and centrifuge at 100,000 × *g* for 2 h (Beckman, Type 70.1 Ti, Fixed angle ultracentrifuge rotor) and the exosomal pellets were suspended in 50 μL PBS.

### Nanoparticle Tracking Analysis (NTA)

NTA measurements were performed using a NanoSight NS500 instrument (NanoSight NTA 3.0 Nanoparticle Tracking and Analysis Release Version Build 0064) as previously described [32, 33].

### Western blot analysis and transmission electron microscopy

Exosomes were solubilized in RIPA buffer (Sigma–Aldrich) and separated by polyacrylamide gel electrophoresis, transferred to a polyvinylidene fluoride (PVDF) membrane (Bio-Rad) and probed with primary rabbit polyclonal antibody anti-CD63 (1:1000; EXOAB-CD63A-1, System Biosciences) and TSG101 (1:500; sc-6037, Santa Cruz Biotechnology). For electron microscopy analysis, exosome pellets were fixed in 3 % (w/v) glutaraldehyde and analyzed under an FEI Tecnai 12 transmission electron microscope (FEI, Hillsboro, Oregon, USA).

### Proteomic Analysis of Endometrial Exosomes by Mass Spectrometry (MS)

Exosomes (10 μg of protein) were solubilized in RIPA buffer (Sigma–Aldrich) and separated by polyacrylamide gel electrophoresis. The gel was fixed in fixing solution (10:1:9; ethanol, acetic acid, MilliQ water respectively) for 15 min, washed in (1:1, ethanol and MilliQ water) for 10 min and washed three times with MilliQ water. Proteins were stained with Coomassie Brilliant Blue R-250 staining solution (Bio-Rad) for 1 h and the gel was allowed to destain in MilliQ water until a clear background was obtained.

In-gel digestion methods for the mass spectrometric identification of exosomal proteins were performed by modification of previously published method [34]. In brief, each sample lane was cut into 24 gel slices and destained twice with 200 mM ammonium bicarbonate in 50 % acetonitrile solution for 45 min at 37 °C, desiccated using a vacuum centrifuge and then resuspended with 20 mM dithiothreitol (DTT) in 25 mM ammonium bicarbonate solution and reduced for 1 h at 65 °C. DTT was then removed, and the samples were alkylated in 50 mM iodoacetamide and 25 mM ammonium bicarbonate at 37 °C in darkness for 40 min. Gel slices were washed three times for 45 min in 25 mM ammonium bicarbonate and then desiccated. Individual dried slices were then allowed to swell in 20 μL of 40 mM ammonium bicarbonate, 10 % acetonitrile containing 20 μg/mL trypsin (Sigma) for 1 h at room temperature. An additional 50 μL of the same solution was added and the samples were incubated overnight at 37 °C.

The supernatants were removed from the gel slices, and residual peptides were washed from the slices by incubating them three times in 50 μL of 0.1 % formic acid for 45 min at 37 °C. The original supernatant and washes were combined and desalted according to a modified version of the stage tip protocol that we have published [35, 36] using a 3-mm piece of an Empore C18 (Octadecyl) SPE Extraction Disk and the eluted peptides were dried in a vacuum centrifuge prior to spectral acquisition.

The digested protein samples were analysed using the TripleTOF® 5600 mass spectrometer (ABSciex, Redwood City, CA) and Eksigent 1D+ NanoLC system with the cHiPLC system to obtain initial high mass accuracy survey MS/MS data, identifying the peptides present in the samples. The ChromXP C18-CL TRAP cHiPLC (200 μm × 6 mm, 3 μm, 120 Å) and analytical cHiPLC columns (200 μm × 15 cm; 3 μm, 120 Å) (Eksigent, Redwood City, CA) were used to separate the digested proteins. A 10 μL aliquot of digested material was injected onto the column and separated with a linear gradient of 5 to 10 % Buffer B for 2 min (Buffer A: 0.1 % Formic acid/water; Buffer B: acetonitrile/0.1 % formic acid), 10 to 40 % Buffer B (58 min), 40 to 50 % Buffer B (10 min), 50 to 95 % (10 min) with a flow rate of 500 nL/min. The column was flushed at 95 % buffer B for 15 min and re-equilibrated with 5 % Buffer B for 6 min. The in-depth proteomic analysis was performed using the Information Dependent Acquisition (IDA) experiments on the TripleTOF® 5600 System interfaced with a nanospray source. The source parameters were as follows: Cur gas at 25 psi, GS1 at 5 psi and IHT at 150 °C. A 250 msec accumulation time was set for the TOFMS survey scan and from this scan, the 10 most intense precursor ions were selected automatically for the MS/MS analysis (accumulation time of 150 msecs per MS/MS scan). Ions were isolated using unit resolution of the quadrupoles and rolling collision energy equation was used to calculate the collision energies of precursors. The precursor selection criteria included a minimum intensity of 50 counts per second (cps) and a charge state greater than 2 + .

Protein identification was determined using the ProteinPilot™ Software (v4.5 beta, AB Sciex, Redwood City, CA) with the Paragon algorithm. The search parameters were as follows: sample type, identification; cys alkylation, iodoacetamide; digestion, Trypsin; Instrument, TripleTOF 5600; special factors, none; and ID focus, biological modifications. The database was downloaded from the UniProt website in October 2015, which contained all proteins from *Bos taurus*. False discovery rate (FDR) was selected in the method and determined using a reversed sequence database. Data were subjected to ontology and pathway analysis using the protein analysis through evolutionary relationships tool (PANTHER) and gene ontology algorithms and classified based on biological process and molecular function categories [37].

### Statistical analyses

The effects of oxygen tensions on ICAR cells are presented as mean ± SE for migration, proliferation and apoptosis assays (*n* = 6 independent experiments in duplicate). The number of exosomes is presented as number of particles per mL (mean ± SE, *n* = 3 independent isolations from 80 million cells each). The effects of oxygen tension on ICAR cells were identified by Student’s T tests (two-tailed) to compare the effect of hypoxia (i.e. 1 % O_2_) with the control group (i.e. 8 % O_2_) using a commercially-available software package (Prism 6, GraphPad Inc, La Jolla, CA 92037 USA).

## Results

### The Effect of Oxygen Tension on Bovine Endometrial (ICAR) cell migration and proliferation

**Fig. 1:**
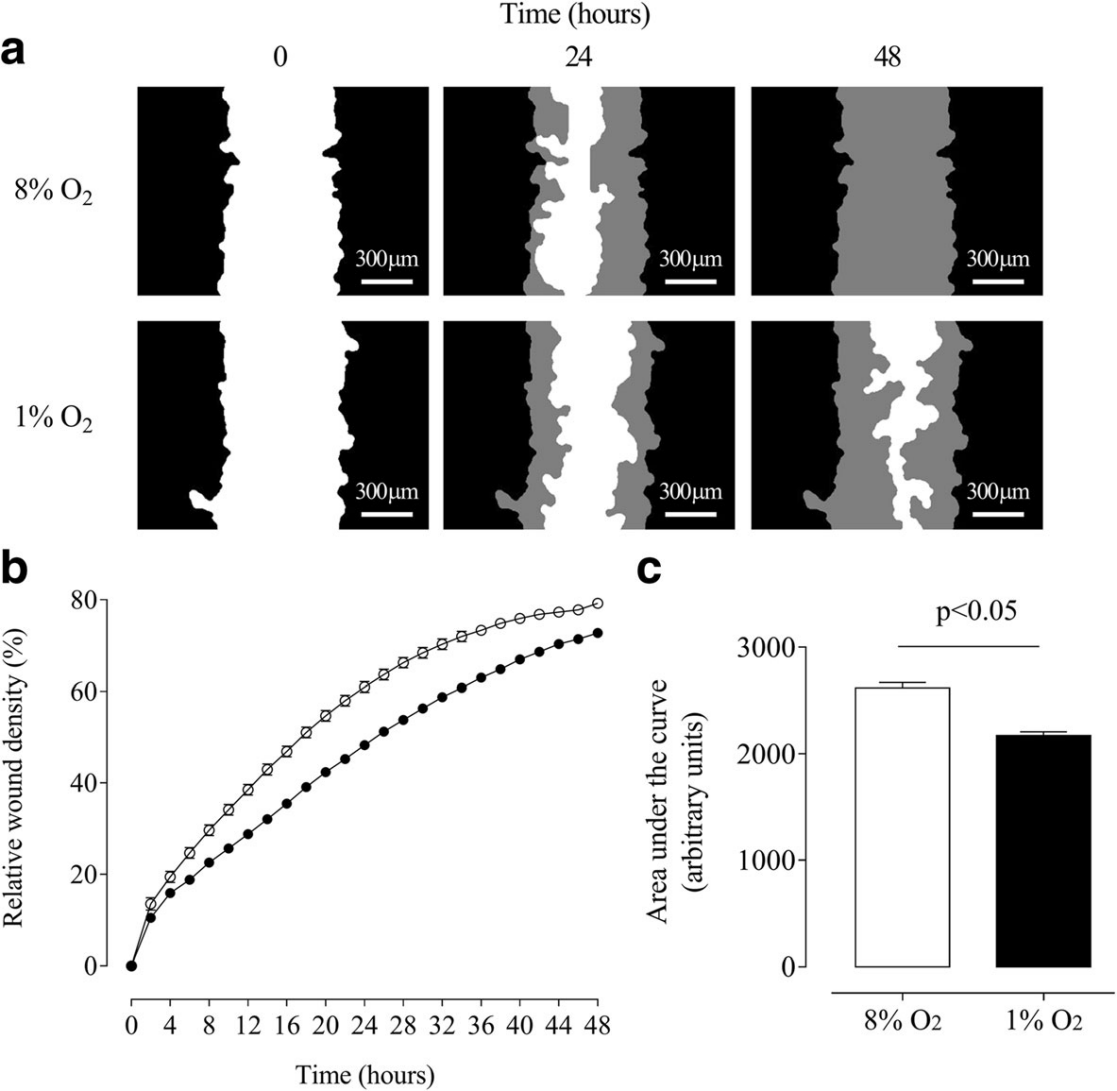
The effects of different oxygen tension on migration of bovine endometrial stromal cells (ICAR). **a** Graphical representation of the initial wound width (*white*) at 0 h and the area of the initial wound covered by advancing cells (*grey*) at 24 h and 48 h, Scale bar 300 μm. **b** Decreased ICAR cell migration under hypoxic conditions (1 % O_2_ (●) compared with a normoxic 8 % O_2_ (○)) over a period of 48 h. **c** Area under the curve analysis from (**b**); 8 % O_2_ (*white bar*) and 1 % O_2_ (*black bar*). Data are presented as mean ± SE, *n* = 6. In (**b**) and (**c**) *P* < 0.05

**Fig. 2:**
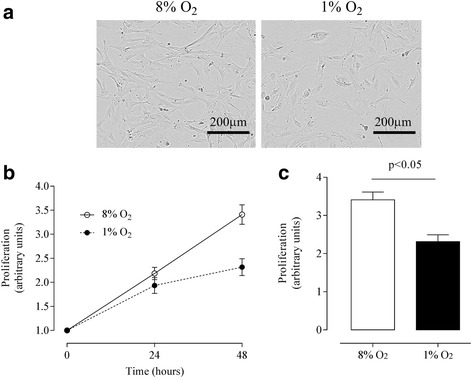
The effects of different oxygen tension on proliferation of bovine endometrial stromal cells (ICAR). **a** Representative phase-contrast image of ICAR cells at 48 h when cultured under hypoxic conditions (1 % O_2_) compared with a normoxic 8 % O_2_, Scale bar 200 μm. **b** Decreased (*p* < 0.01) ICAR cell proliferation under hypoxic conditions (1 % O_2_ (●)) compared with a normoxic 8 % O_2_ (○) over a period of 48 h. **c** Area under the curve analysis from (**b**); 8 % O_2_ (*white bar*) and 1 % O_2_ (*black bar*). Data are presented as mean ± SE, *n* = 6. In (C) *P* < 0.05

The effect of normal oxygen tension (i.e. 8 % O_2_) and hypoxia (i.e. 1 % O_2_) on ICAR cell migration is presented in Fig. 1. ICAR cell migration was significantly lower under hypoxia compared with normal oxygen tension (Fig. 1a). Hypoxia decreased ICAR cell migration in a time-dependent manner (Fig. 1b). Area under the curve analysis indicated that hypoxia decreased ICAR cell migration by ~20 % compared with values observed at 8 % O_2_ (2173 ± 36 and 2620 ± 50 for 1 % O_2_ and 8 % O_2_, respectively) (Fig. 1c). Interestingly, hypoxia decreased ICAR cell proliferation in a time-dependent manner (Fig. 2a and b). Area under curve analysis showed that at 1 % O_2_, the proliferative capacity of ICAR cells was inhibited (*p* < 0.05) ~32 % compared with cell proliferation at 8 % O_2_ (2.32 ± 0.18 and 3.41 ± 0.2 for 1 % O_2_ and 8 % O_2_, respectively) (Fig. 2c).

### The Effect of Oxygen Tension on Bovine Endometrial (ICAR) cell apoptosis

**Fig. 3:**
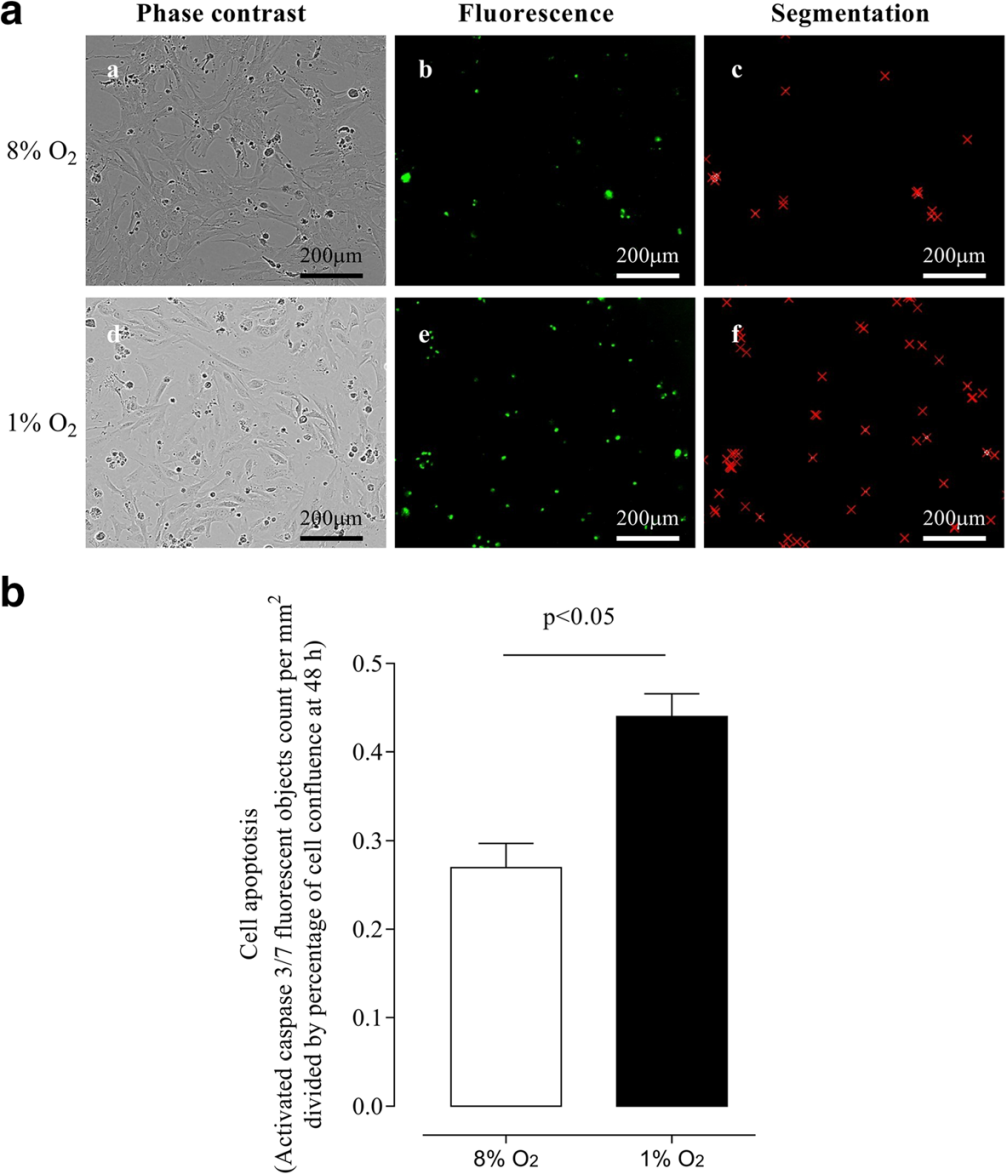
The effects of different oxygen tension on activation of apoptotic protein caspase-3 of bovine endometrial stromal cells (ICAR). ICAR cells were cultured under normoxic (8 % O_2_) or hypoxic (1 % O_2_) conditions and the activated caspase-3/7 fluorescence was measured at 48 h. **A** Representative phase-contrast images (a and d), fluorescent signal images (b and e) and acquired fluorescent signal using integrated object counting algorithm with IncuCyte™ (Segmentation; c and f), Scale bar 400 μm. **B** Increased apoptosis of ICAR cells under hypoxic conditions as determined by acquired fluorescent signal using integrated object counting algorithm with IncuCyte™ were normalized against cell confluence, 8 % O_2_ (*white bar*) and 1 % O_2_ (black bar). Data are presented as mean ± SE, *n* = 6. In (B) *P* < 0.05

The effect of oxygen tension on cell apoptosis is presented in Fig. 3. A hypoxic (1 % O_2_) environment altered cell morphology compared with cells cultured under normal conditions (8 % O_2_), displaying morphological hallmarks of apoptotic death (Fig. 3A ,a and d). Fluorescent images acquired with IncuCyte™ (Fig. 3A, b and e) showed greater fluorescence in cells cultured under 1 % O_2_, indicating a higher activation of caspase-3/7 under hypoxic conditions compared with 8 % O_2_ (Fig. 3A, b and e). Apoptosis was quantified using the object counting algorithm in which the number of fluorescent objects was indicated with red x’s in Fig. 3A (c and f). Quantification analysis showed that hypoxia increased (~1.6 fold) the apoptosis ratio (presented as activated caspase 3/7 fluorescent objects count per mm2 divided by percentage of cell confluence at 48 h) compared with cells cultured under normal oxygen tension (Fig. 3B).

### The Effect of Oxygen Tension on Exosome Release from Bovine Endometrial Cells (ICAR)

**Fig. 4:**
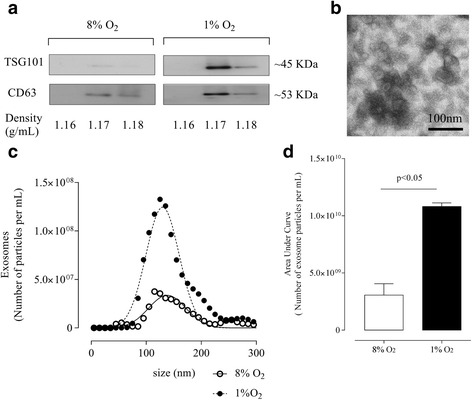
Characterization of exosomes release from 8 % O_2_ and 1 % O_2_ ICAR cell-conditioned media. Exosomes were characterized after enrichment from the 100,000 x *g* pellet by buoyant density centrifugation (see Methods). **a** Representative Western blot for exosome markers: TSG101 and CD63. **b** Representative electron micrograph exosome fractions, Scale bar 100 nm. **c** Representative Nanosight measurement of particle-size distribution exosomes from 8 % O_2_ and 1 % O_2_ cell-conditioned media after buoyant density gradient ultracentrifugation. (8 % normoxic condition mean size (127 ± 1.7 nm) (○), 1 % hypoxic condition mean size (123 ± 2.7 nm) (●) over a period of 48 h). **d** Exosomes concentration presented as vesicle per million cells per 48 h was higher (*p* < 0.05) at hypoxia (1 % O_2_) compared to normal oxygen tension (8 % O_2_); 8 % O_2_ (*white bar*) and 1 % O_2_ (black bar). Data are presented as mean ± SE, *n* = 3

Exosomes were enriched by buoyant density gradient (see Material and Methods). We fractioned the 100,000 × *g* pellet into 12 fractions and the Western blot analysis for TSG101 and CD63 showed positive protein abundance in fractions 1.17 and 1.18 g/mL (Fig. 4a). Exosomes were pooled between densities 1.16 and 1.18 g/mL. Morphology of exosomes was determined by electron microscopy (Fig. 4b), exosomes displayed a cup-shaped morphology with an estimated diameter of 100 nm. Hypoxia did not alter the size distribution of exosomes compared with normal oxygen tension (123 ± 2.7 nm versus 127 ± 1.7 nm for 8 % O_2_ and 1 % O_2_, respectively) (Fig. 4c). Interestingly, hypoxia increased (~3.6 fold) the number of exosomes compared with values observed at normal oxygen tension (Fig. 4d).

### Proteomic Analysis of Bovine Endometrial ICAR-Derived Exosomes

**Fig. 5:**
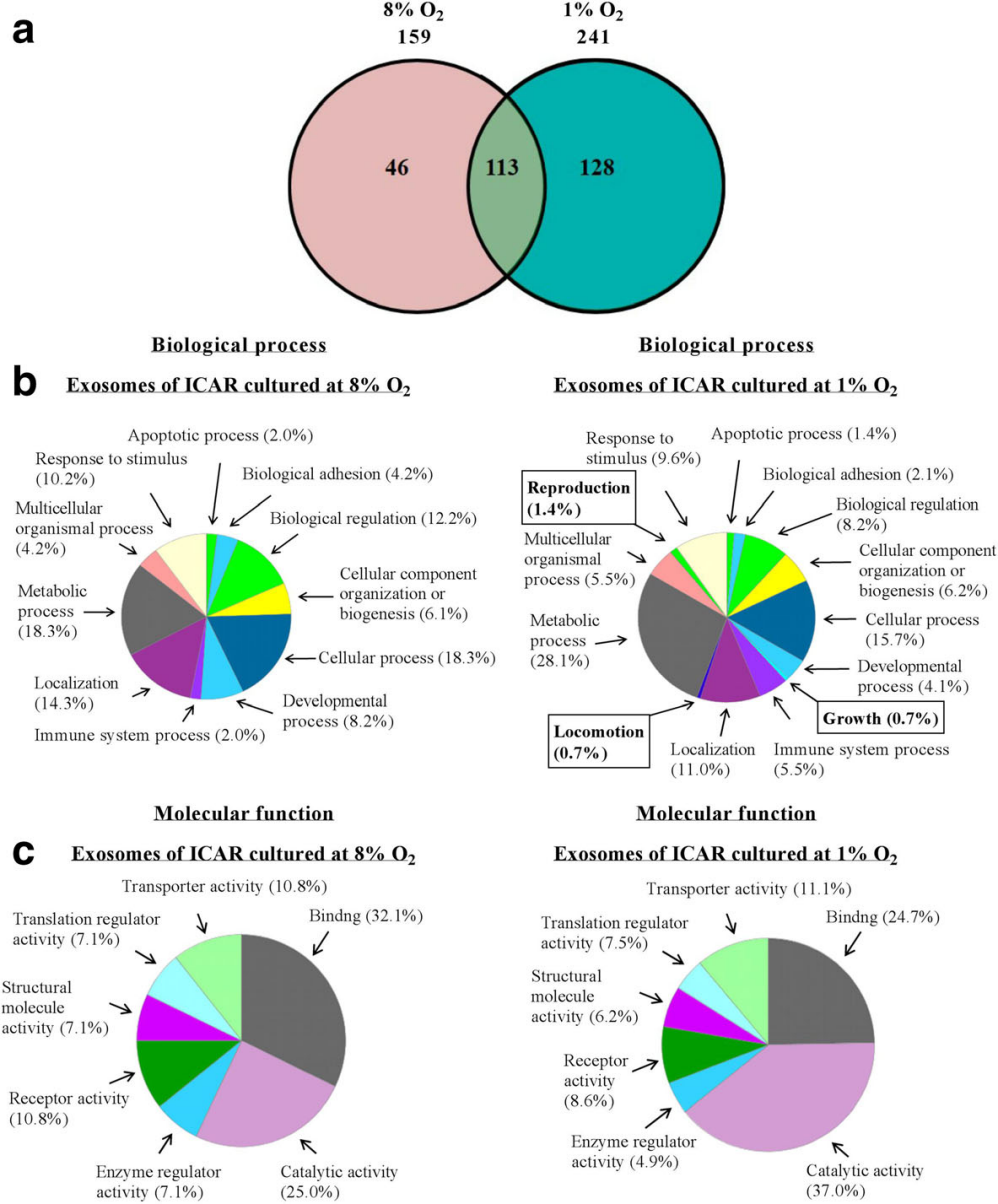
Proteomic analysis of bovine endometrial ICAR-derived exosomes. Mass spectrometric analyses of ICAR cell-derived exosome proteins. **a** Representative Venn diagram of common and unique proteins identified by 5600 Triple TOF MS (ABSciex) from exosomes released by ICAR cells at 48 h at both 8 % O_2_ and 1 % O_2_. **b** The gene ontology classification of ICAR cell-derived exosome proteins, on the basis of their involvement in biological process, identified clusters that are unique to and present only in exosomes of ICAR cultured at 1 % O_2_ but not those at 8 % O_2_. These biological processes were: growth (0.7 %), locomotion (0.7 %) and reproduction (1.4 %). **c** Molecular function (using PANTHER and Gene Ontology algoritnms) of exosome proteins were mostly related to binding and catalytic activity in both ICAR cultured at 1 % O_2_ and at 8 % O_2_

Mass spectrometric analysis identified over 250 exosomal proteins with 113 similar proteins identified as present in both exosomes of ICAR cultured at 1 % O_2_ and at 8 % O_2_ 128 proteins identified as unique to exosomes of ICAR cultured at 1 % O_2_; 46 proteins were identified as unique to exosomes of ICAR cultured at 8 % O_2_ (Table 1 A-C; Fig. 5a). Data were subjected to ontology and pathway analysis using PANTHER and gene ontology algorithms and classified based on biological process (Fig. 5b) and molecular function (Fig. 5c). In biological process, the clusters identified from individual proteins that are unique to and present only in exosomes of ICAR cultured at 1 % O_2_ but not those at 8 % O_2_ were: growth (0.7 %), locomotion (0.7 %) and reproduction (1.4 %) (Fig. 5b). In molecular functions, the proteins related to binding and catalytic activity were the greatest recognized in both exosomes of ICAR cultured at 1 % O_2_ and to those of ICAR cultured at 8 % O_2_ (Fig. 5c).

**Table 1:** List of the common proteins identified in exosomes of ICAR cultured at 1 % O_2_ and at 8 % O_2_

A. List of 113 common proteins identified in exosomes of ICAR cultured at 1 % O_2_ and at 8 % O_2_				
Protein ID	Name	Gene Name	Biological Process (Total # Gene 69; Total #Function 146)	Molecular function (Total # Gene 69; Total #Function 81)
A1L523_BOVIN	Copine II (Fragment)	CPNE2		
A3KN51_BOVIN	TSG101 protein	TSG101	Metabolic process	Catalytic activity
A5D7L1_BOVIN	CLEC11A protein	CLEC11A	Cellular process/Developmental process	Binding/Structural molecule activity
A5D9D2_BOVIN	Complement component 4 binding protein, alpha chain	C4BPA		
A5PJ69_BOVIN	SERPINA10 protein	SERPINA10	Biological regulation/Metabolic process	Catalytic activity/Enzyme regulator activity
A5PJE3_BOVIN	Fibrinogen alpha chain	FGA		
A5PK77_BOVIN	SERPINA11 protein	SERPINA11	Biological regulation/Metabolic process	Catalytic activity/Enzyme regulator activity
A6QLB7_BOVIN	Adenylyl cyclase-associated protein	CAP1		
A6QLL8_BOVIN	Fructose-bisphosphate aldolase	ALDOA		
A6QNZ7_BOVIN	Keratin 10 (Epidermolytic hyperkeratosis; keratosis palmaris et plantaris)	KRT10		
A6QPP2_BOVIN	SERPIND1 protein	SERPIND1	Biological regulation/Metabolic process	Catalytic activity/Enzyme regulator activity
A6QPR1_BOVIN	PCYOX1 protein	PCYOX1		
LG3BP_BOVIN	Galectin-3-binding protein	LGALS3BP	Apoptotic process/Biological adhesion/Biological regulation/Cellular process/Developmental process/Immune system process/localization/Metabolic process	Catalytic activity/Receptor activity
A7MB82_BOVIN	C1QTNF3 protein	C1QTNF3		
A7YWB6_BOVIN	LOC539596 protein	LOC539596		
B0JYM4_BOVIN	Tetraspanin	CD63		
B0JYN6_BOVIN	Alpha-2-HS-glycoprotein	AHSG		
B0JYQ0_BOVIN	ALB protein	ALB		
B5B3R8_BOVIN	Alpha S1 casein	CSN1S1		
E1BDG5_BOVIN	Protein Wnt	WNT5A	Biological regulation/Cellular process/Developmental process/Multicellular organismal process/Response to stimulus	Binding
CBG_BOVIN	Corticosteroid-binding globulin	SERPINA6	Biological regulation/Metabolic process	Catalytic activity/Enzyme regulator activity
F1MAV0_BOVIN	Fibrinogen beta chain	FGB		
F1MB08_BOVIN	Alpha-enolase	ENO1		
F1MC11_BOVIN	Keratin, type I cytoskeletal 14	KRT14		
F1MM32_BOVIN	Sulfhydryl oxidase	QSOX1		Catalytic activity
F1MMK9_BOVIN	Protein AMBP	AMBP		
F1MMP5_BOVIN	Inter-alpha-trypsin inhibitor heavy chain H1	ITIH1		
ITA3_BOVIN	Integrin alpha-3	ITGA3		
F1MNW4_BOVIN	Inter-alpha-trypsin inhibitor heavy chain H2	ITIH2		
F1MSZ6_BOVIN	Antithrombin-III	SERPINC1		
F1MTV5_BOVIN	Amino acid transporter	SLC1A5		
F1MW44_BOVIN	Coagulation factor XIII A chain	F13A1		
F1MXJ5_BOVIN	IST1 homolog	IST1		
F1MXX6_BOVIN	Lactadherin	MFGE8		
F1MY85_BOVIN	Complement C5a anaphylatoxin	C5		
F1N045_BOVIN	Complement component C7	C7		
HTRA1_BOVIN	Serine protease HTRA1	HTRA1	Cellular process/Metabolic process	Catalytic activity
F1N1I6_BOVIN	Gelsolin	GSN		
F6QVC9_BOVIN	Annexin	ANXA5		
G3X6N3_BOVIN	Serotransferrin	TF		
G5E5A9_BOVIN	Fibronectin	FN1		
G5E5V0_BOVIN	Carboxypeptidase N catalytic chain	CPN1		
G8JKX6_BOVIN	Tetraspanin (Fragment)	CD9		
I7CT57_BOVIN	Vitamin D binding protein			
M0QVZ6_BOVIN	Keratin, type II cytoskeletal 5	KRT5		
THRB_BOVIN	Prothrombin	F2	Immune system process/Metabolic process/Response to stimulus	Catalytic activity
PROC_BOVIN	Vitamin K-dependent protein C (Fragment)	PROC	Response to stimulus	Binding
KNG2_BOVIN	Kininogen-2	KNG2		
THYG_BOVIN	Thyroglobulin	TG	Metabolic process	Catalytic activity
HBA_BOVIN	Hemoglobin subunit alpha	HBA	localization/Multicellular organismal process	
HBBF_BOVIN	Hemoglobin fetal subunit beta		localization/Multicellular organismal process	
ALBU_BOVIN	Serum albumin	ALB	localization	
ANXA2_BOVIN	Annexin A2	ANXA2	Developmental process/Metabolic process	
ASSY_BOVIN	Argininosuccinate synthase	ASS1	Cellular process/Metabolic process	Catalytic activity
APOH_BOVIN	Beta-2-glycoprotein 1	APOH	Cellular process/Immune system process/localization/Metabolic process/Response to stimulus	Catalytic activity/Receptor activity/Transporter activity
CLUS_BOVIN	Clusterin	CLU		
HSP7C_BOVIN	Heat shock cognate 71 kDa protein	HSPA8	Cellular component organization or biogenesis/Immune system process/Metabolic process/Response to stimulus	
ANXA7_BOVIN	Annexin A7	ANXA7	Metabolic process	
ANX11_BOVIN	Annexin A11	ANXA11	Metabolic process	
A2AP_BOVIN	Alpha-2-antiplasmin	SERPINF2	Biological regulation/Metabolic process	Catalytic activity/Enzyme regulator activity
A1AT_BOVIN	Alpha-1-antiproteinase	SERPINA1	Biological regulation/Metabolic process	Catalytic activity/Enzyme regulator activity
GDIB_BOVIN	Rab GDP dissociation inhibitor beta	GDI2	Biological regulation/Cellular process/localization/Metabolic process/Multicellular organismal process	Binding/Catalytic activity/Enzyme regulator activity
F12AI_BOVIN	Factor XIIa inhibitor			
ITB1_BOVIN	Integrin beta-1	ITGB1	Biological adhesion/Cellular process/Response to stimulus	Receptor activity
ITIH3_BOVIN	Inter-alpha-trypsin inhibitor heavy chain H3	ITIH3	Biological regulation/Metabolic process	Binding/Catalytic activity/Enzyme regulator activity
ACTB_BOVIN	Actin, cytoplasmic 1	ACTB	Cellular component organization or biogenesis/Cellular process/Developmental process/localization	Structural molecule activity
ANXA6_BOVIN	Annexin A6	ANXA6	Metabolic process	
CFAB_BOVIN	Complement factor B	CFB	Biological adhesion/Cellular process/Immune system process/localization/Metabolic process/Response to stimulus	Catalytic activity/Receptor activity/Transporter activity
TBA1B_BOVIN	Tubulin alpha-1B chain		Cellular process/Developmental process/localization	Structural molecule activity
LUM_BOVIN	Lumican	LUM	Biological adhesion/Biological regulation/Cellular process/Developmental process/Immune system process/Metabolic process/Multicellular organismal process	Receptor activity
UPAR_BOVIN	Urokinase plasminogen activator surface receptor	PLAUR		
5NTD_BOVIN	5’-nucleotidase	NT5E	Metabolic process	Catalytic activity
PGM1_BOVIN	Phosphoglucomutase-1	PGM1	Cellular process/Metabolic process	Catalytic activity
Q09TE3_BOVIN	Insulin-like growth factor binding protein acid labile subunit			
Q17R18_BOVIN	Adenosine kinase	ADK		
FA5_BOVIN	Coagulation factor V	F5	Biological adhesion/Biological regulation/Cellular process/Developmental process/Immune system process/localization/Metabolic process/Multicellular organismal process/Response to stimulus	Binding/Catalytic activity/Enzyme regulator activity/Receptor activity/Transporter activity
Q2KIF2_BOVIN	Leucine-rich alpha-2-glycoprotein 1	LRG1	Cellular process/Multicellular organismal process	Receptor activity
CBPB2_BOVIN	Carboxypeptidase B2	CPB2	Metabolic process	Catalytic activity
Q2KJ47_BOVIN	EH-domain containing 2	EHD2	Biological regulation/Cellular process/localization/Metabolic process/Multicellular organismal process	Binding/Catalytic activity/Enzyme regulator activity
TBB5_BOVIN	Tubulin beta-5 chain	TUBB5	Cellular process/Developmental process/localization	Structural molecule activity
A1BG_BOVIN	Alpha-1B-glycoprotein	A1BG	Cellular process/Immune system process/Response to stimulus	Binding/Receptor activity
HPT_BOVIN	Haptoglobin	HP	Biological regulation/Immune system process/localization/Metabolic process/Multicellular organismal process/Reproduction/Response to stimulus	Binding/Catalytic activity/Enzyme regulator activity/Receptor activity
CO3_BOVIN	Complement C3	C3	Biological regulation/Cellular process/Metabolic process/Response to stimulus	Binding/Catalytic activity/Enzyme regulator activity
Q3MHH8_BOVIN	Alpha-amylase	AMY2A		
SAHH_BOVIN	Adenosylhomocysteinase	AHCY	Cellular process/Metabolic process	Catalytic activity
CO9_BOVIN	Complement component C9	C9	Cellular process/localization/Metabolic process/Response to stimulus	Catalytic activity/Receptor activity/Transporter activity
Q3MHW2_BOVIN	F10 protein (Fragment)	F10		
Q3MHZ0_BOVIN	FLOT1 protein (Fragment)	FLOT1		
Q3SYR0_BOVIN	Serpin peptidase inhibitor, clade A (Alpha-1 antiproteinase, antitrypsin), member 7	SERPINA7		
FETA_BOVIN	Alpha-fetoprotein	AFP	Developmental process/localization	
Q3SZH5_BOVIN	Angiotensinogen	AGT		
HEMO_BOVIN	Hemopexin	HPX	localization	
Q3SZZ9_BOVIN	FGG protein	FGG		
PGK1_BOVIN	Phosphoglycerate kinase 1	PGK1	Metabolic process	Catalytic activity
Q3T101_BOVIN	IGL@ protein	IGL@		
G6PI_BOVIN	Glucose-6-phosphate isomerase	GPI	Metabolic process	Catalytic activity
Q3ZBX0_BOVIN	Basigin	BSG		
Q3ZC87_BOVIN	Pyruvate kinase (Fragment)	PKM2		
Q3ZCI4_BOVIN	6-phosphogluconate dehydrogenase, decarboxylating	PGD	Metabolic process	Catalytic activity
FETUB_BOVIN	Fetuin-B	FETUB		
EHD1_BOVIN	EH domain-containing protein 1	EHD1	Biological regulation/Cellular process/localization/Metabolic process/Multicellular organismal process	Binding/Catalytic activity/Enzyme regulator activity
HPPD_BOVIN	4-hydroxyphenylpyruvate dioxygenase	HPD	Metabolic process	Catalytic activity
Q5EA67_BOVIN	Inter-alpha (Globulin) inhibitor H4 (Plasma Kallikrein-sensitive glycoprotein)	ITIH4		
Q5GN72_BOVIN	Alpha-1-acid glycoprotein	agp		
BHMT1_BOVIN	Betaine--homocysteine S-methyltransferase 1	BHMT	Cellular process/Metabolic process	Catalytic activity
Q5J801_BOVIN	Endopin 2B			
Q6T182_BOVIN	Sex hormone-binding globulin (Fragment)	SHBG		
A2MG_BOVIN	Alpha-2-macroglobulin	A2M	Biological regulation/Cellular process/Immune system process/Metabolic process/Response to stimulus	Binding/Catalytic activity/Enzyme regulator activity
PEDF_BOVIN	Pigment epithelium-derived factor	SERPINF1	Biological regulation/Metabolic process	Catalytic activity/Enzyme regulator activity
CHIA_BOVIN	Acidic mammalian chitinase	CHIA	Immune system process/Metabolic process/Response to stimulus	Binding/Catalytic activity
IPSP_BOVIN	Plasma serine protease inhibitor	SERPINA5	Biological regulation/Metabolic process	Catalytic activity/Enzyme regulator activity
SPA31_BOVIN	Serpin A3-1	SERPINA3-1	Biological regulation/Metabolic process	Catalytic activity/Enzyme regulator activity
V6F9A2_BOVIN	Apolipoprotein A-I preproprotein	APOA1		
B. List of 128 unique proteins identified in exosomes of ICAR cultured at 1 % O_2_				
Protein ID	Name	Gene Name	Biological Process (Total # Gene 22; Total #Function 49)	Molecular function (Total # Gene 22; Total #Function 28)
G3X6T9_BOVIN	Flotillin-2 (Fragment)	FLOT2		
TSP1_BOVIN	Thrombospondin-1	THBS1		
F1N2L9_BOVIN	4-trimethylaminobutyraldehyde dehydrogenase	ALDH9A1		
E1B9F6_BOVIN	Elongation factor 1-alpha	EEF1A1		
APOE_BOVIN	Apolipoprotein E	APOE	Apoptotic process/Biological regulation/Cellular component organization or biogenesis/Cellular process/Developmental process/Growth/localization/Metabolic process/Multicellular organismal process/Response to stimulus	Binding/Catalytic activity/ Enzyme regulator activity/Transporter activity
G1K1R6_BOVIN	Galactokinase	GALK1		
G3P_BOVIN	Glyceraldehyde-3-phosphate dehydrogenase	GAPDH	Metabolic process	Catalytic activity
Q0P5B0_BOVIN	Arrestin domain containing 1	ARRDC1		
RL40_BOVIN	Ubiquitin-60S ribosomal protein L40	UBA52	Metabolic process	Binding/Structural molecule activity
A5D9B6_BOVIN	Syntenin	SDCBP		
Q8HZY1_BOVIN	Serine protease inhibitor clade E member 2	SERPINE2		
Q5E962_BOVIN	Aldo-keto reductase family 1, member B1	AKR1B1		
A7MBH9_BOVIN	GNAI2 protein	GNAI2	Biological regulation/Cellular process/Metabolic process/Response to stimulus	Binding/Catalytic activity
GBB2_BOVIN	Guanine nucleotide-binding protein G(I)/G(S)/G(T) subunit beta-2	GNB2	Cellular process/Metabolic process/Multicellular organismal process	Binding/Catalytic activity
I6YIV1_BOVIN	Annexin			
F16P1_BOVIN	Fructose-1,6-bisphosphatase 1	FBP1	Metabolic process	
F1N3Q7_BOVIN	Apolipoprotein A-IV	APOA4		
AK1A1_BOVIN	Alcohol dehydrogenase [NADP(+)]	AKR1A1	localization/Metabolic process	Catalytic activity/Transporter activity
A5D784_BOVIN	CPNE8 protein	CPNE8	localization	
HS90A_BOVIN	Heat shock protein HSP 90-alpha	HSP90AA1	Immune system process/Metabolic process/Response to stimulus	
Q1JPA2_BOVIN	Eukaryotic translation elongation factor 1 gamma (Fragment)	EEF1G		
SERA_BOVIN	D-3-phosphoglycerate dehydrogenase	PHGDH	Metabolic process	Catalytic activity
Q3T085_BOVIN	OGN protein	OGN		
A8DBT6_BOVIN	Monocyte differentiation antigen CD14	CD14		
A5PK73_BOVIN	Fructose-bisphosphate aldolase	ALDOB		
G5E5U7_BOVIN	S-adenosylmethionine synthase	MAT1A		
F1N2W0_BOVIN	Prostaglandin reductase 1	PTGR1		
IF4A1_BOVIN	Eukaryotic initiation factor 4A-I	EIF4A1	Biological regulation/Metabolic process	Binding/Catalytic activity/Translation regulator activity
Q05B55_BOVIN	IGK protein	IGK		
F1N1D4_BOVIN	Protein tweety homolog	TTYH3	localization	Transporter activity
A4FV94_BOVIN	KRT6A protein	KRT6A		
RGN_BOVIN	Regucalcin	RGN	Cellular process/localization/Metabolic process	Binding/Catalytic activity
1433E_BOVIN	14-3-3 protein epsilon	YWHAE	Cellular process	
Q2HJB6_BOVIN	Procollagen C-endopeptidase enhancer	PCOLCE	Biological adhesion/Biological regulation/Cellular process/Developmental process/Immune system process/localization/Metabolic process/Multicellular organismal process/Response to stimulus	Binding/Catalytic activity/Enzyme regulator activity/Receptor activity/Transporter activity
B8YB76_BOVIN	Homogentisate 1,2-dioxygenase	HGD		
DHSO_BOVIN	Sorbitol dehydrogenase	SORD	Metabolic process	Catalytic activity
HS71A_BOVIN	Heat shock 70 kDa protein 1A	HSPA1A	Cellular component organization or biogenesis/Immune system process/Metabolic process/Response to stimulus	
Q3ZBQ9_BOVIN	APOM protein	APOM		
PYGL_BOVIN	Glycogen phosphorylase, liver form	PYGL	Metabolic process	Catalytic activity
A6QP30_BOVIN	CPN2 protein	CPN2	Cellular process/Multicellular organismal process	Receptor activity
ARF3_BOVIN	ADP-ribosylation factor 3	ARF3	Cellular process/localization/Metabolic process	Binding/Catalytic activity
G3MYH4_BOVIN	Tetraspanin (Fragment)	CD81		
ACTC_BOVIN	Actin, alpha cardiac muscle 1	ACTC1	Cellular component organization or biogenesis/Cellular process/Developmental process/localization	Structural molecule activity
GALM_BOVIN	Aldose 1-epimerase	GALM	Metabolic process	Catalytic activity
TSN6_BOVIN	Tetraspanin-6	TSPAN6	Biological adhesion/Cellular process/Immune system process/Multicellular organismal process/Reproduction/Response to stimulus	Binding/Receptor activity
Q3ZC83_BOVIN	Solute carrier family 29 (Nucleoside transporters), member 1	SLC29A1	localization/Metabolic process	Transporter activity
B4GA1_BOVIN	Beta-1,4-glucuronyltransferase 1	B4GAT1	Metabolic process	Catalytic activity
ADA10_BOVIN	Disintegrin and metalloproteinase domain-containing protein 10	ADAM10	Apoptotic process/Developmental process/Reproduction	
A6QR28_BOVIN	Phosphoserine aminotransferase	PSAT1	Metabolic process	Catalytic activity
Q1JPB6_BOVIN	Acetyl-Coenzyme A acetyltransferase 2	ACAT2		
DDBX_BOVIN	Dihydrodiol dehydrogenase 3		localization/Metabolic process	Catalytic activity/Transporter activity
A2VE11_BOVIN	IGSF8 protein	IGSF8		
F1MS32_BOVIN	Apolipoprotein D	APOD		
A6QP64_BOVIN	VPS37B protein (Fragment)	VPS37B		
Q2KIW4_BOVIN	Lecithin-cholesterol acyltransferase	LCAT	Metabolic process	Catalytic activity
GBB1_BOVIN	Guanine nucleotide-binding protein G(I)/G(S)/G(T) subunit beta-1	GNB1	Cellular process/Metabolic process	Binding/Catalytic activity
GNA11_BOVIN	Guanine nucleotide-binding protein subunit alpha-11	GNA11	Biological regulation/Cellular process/Metabolic process/Response to stimulus	Catalytic activity
Q17QK4_BOVIN	Epoxide hydrolase 2, cytoplasmic	EPHX2		
K2C7_BOVIN	Keratin, type II cytoskeletal 7	KRT7	Cellular component organization or biogenesis/Cellular process/Developmental process	Structural molecule activity
CLIC1_BOVIN	Chloride intracellular channel protein 1	CLIC1	Biological regulation/Cellular process/Metabolic process/Response to stimulus	Binding/Catalytic activity/Structural molecule activity/Translation regulator activity
Q08DW4_BOVIN	Mannan-binding lectin serine peptidase 1 (C4/C2 activating component of Ra-reactive factor)	MASP1		
B4GT1_BOVIN	Beta-1,4-galactosyltransferase 1	B4GALT1		
A5D7E6_BOVIN	Tetraspanin	CD82	Cellular process/Response to stimulus	Binding/Receptor activity
A5D973_BOVIN	Alpha isoform of regulatory subunit A, protein phosphatase 2	PPP2R1A		
E1B726_BOVIN	Plasminogen	PLG		
G5E6I9_BOVIN	Histone H2B	LOC101904777	Cellular component organization or biogenesis/Cellular process/Metabolic process	Binding
ADIPO_BOVIN	Adiponectin	ADIPOQ		
F1MBC5_BOVIN	Coagulation factor IX	F9		
A2VDL2_BOVIN	Solute carrier family 2 (Facilitated glucose transporter), member 3	SLC2A3		
VPS4B_BOVIN	Vacuolar protein sorting-associated protein 4B	VPS4B		
G3X8B1_BOVIN	Peptidyl-prolyl cis-trans isomerase	LOC613401		
K4JB97_BOVIN	Alpha-2-macroglobulin variant 4	A2M		
ACTG_BOVIN	Actin, cytoplasmic 2	ACTG1	Cellular component organization or biogenesis/Cellular process/localization	Structural molecule activity
Q1JPG7_BOVIN	Pyruvate kinase	PKLR		
GTR1_BOVIN	Solute carrier family 2, facilitated glucose transporter member 1	SLC2A1		
F1N342_BOVIN	Protein tweety homolog	TTYH2	localization	Transporter activity
ADHX_BOVIN	Alcohol dehydrogenase class-3	ADH5	Metabolic process	Catalytic activity
URP2_BOVIN	Fermitin family homolog 3	FERMT3		
E1B7N2_BOVIN	Histone H4	HIST1H4I	Cellular component organization or biogenesis/Cellular process/Metabolic process	Binding
EF2_BOVIN	Elongation factor 2	EEF2	Biological regulation/Metabolic process	Binding/Translation regulator activity
KLKB1_BOVIN	Plasma kallikrein	KLKB1	Biological regulation/localization/Metabolic process/Response to stimulus	Binding/Catalytic activity/Enzyme regulator activity/Receptor activity
ESTD_BOVIN	S-formylglutathione hydrolase	ESD	Metabolic process	Catalytic activity
SEPR_BOVIN	Prolyl endopeptidase FAP	FAP	Cellular process/Immune system process/localization/Metabolic process/Multicellular organismal process / Response to stimulus	Binding/Catalytic activity
Q5EA54_BOVIN	Solute carrier family 3 (Activators of dibasic and neutral amino acid transport), member 2	SLC3A2		
Q1JPD9_BOVIN	G protein-coupled receptor, family C, group 5, member B	GPRC5B	Cellular process	Receptor activity
F1MS05_BOVIN	Aconitate hydratase	ACO1		
F1MJ12_BOVIN	Complement C1s subcomponent	C1S		
CNDP2_BOVIN	Cytosolic non-specific dipeptidase	CNDP2	Metabolic process	Catalytic activity
Q2TBQ1_BOVIN	Coagulation factor XIII, B polypeptide	F13B	Biological adhesion/Cellular process/Immune system process/localization/Metabolic process/Response to stimulus	Catalytic activity/Receptor activity/Transporter activity
Q1JP72_BOVIN	Colony stimulating factor 1 receptor	CSF1R		
Q0VD03_BOVIN	CD44 antigen	CD44		
G3X6Y4_BOVIN	Osteomodulin	OMD		
GAMT_BOVIN	Guanidinoacetate N-methyltransferase	GAMT		
VWA1_BOVIN	von Willebrand factor A domain-containing protein 1	VWA1		
SERC3_BOVIN	Serine incorporator 3	SERINC3		
Q862H8_BOVIN	Similar to 40S ribosomal protein SA (P40) (Fragment)			
A8E4P3_BOVIN	STOM protein	STOM		
F1MHP6_BOVIN	Adenylosuccinate lyase	ADSL		
E1BMG9_BOVIN	10-formyltetrahydrofolate dehydrogenase	ALDH1L1	Metabolic process	Catalytic activity
Q705V4_BOVIN	Kappa-casein (Fragment)	csn3		
G3X6Q8_BOVIN	Pentraxin-related protein PTX3	PTX3		
K7QEL2_BOVIN	MHC class I antigen	BoLA		
TCPQ_BOVIN	T-complex protein 1 subunit theta	CCT8	Cellular component organization or biogenesis / Metabolic process	
F1N6Z0_BOVIN	26S proteasome non-ATPase regulatory subunit 5	PSMD5		
ARLY_BOVIN	Argininosuccinate lyase	ASL	Metabolic process	Catalytic activity
E1BNG2_BOVIN	alpha-1,2-Mannosidase	MAN1A1	Metabolic process	
F1MU79_BOVIN	Peptidyl-prolyl cis-trans isomerase FKBP4	FKBP4		
DPYL2_BOVIN	Dihydropyrimidinase-related protein 2	DPYSL2	Metabolic process	Catalytic activity
PRS23_BOVIN	Serine protease 23	PRSS23		
B0JYN1_BOVIN	Cathepsin L2	CTSL2		
A4FV99_BOVIN	FCNB protein	FCNB		
A7YW37_BOVIN	CD58 protein (Fragment)	CD58	Immune system process/Response to stimulus	Binding
F1MTP5_BOVIN	WD repeat-containing protein 1	WDR1		
A7E3D0_BOVIN	CCDC45 protein (Fragment)	CCDC45		
Q0VCK1_BOVIN	Myeloid-associated differentiation marker	MYADM		
A1L570_BOVIN	Ephrin-B1	EFNB1	Biological regulation/Cellular component organization or biogenesis/Cellular process/Developmental process/locomotion/Multicellular organismal process/Response to stimulus	Binding
F1N049_BOVIN	Actin-related protein 3 (Fragment)	ACTR3		
PAI1_BOVIN	Plasminogen activator inhibitor 1	SERPINE1	Biological regulation/Metabolic process	Catalytic activity/Enzyme regulator activity
Q3ZC30_BOVIN	Sulfotransferase	SULT1E1		
COL11_BOVIN	Collectin-11	COLEC11	Biological regulation/Immune system process/Multicellular organismal process	
MPZL1_BOVIN	Myelin protein zero-like protein 1	MPZL1	Cellular process/localization	Transporter activity
G5E595_BOVIN	Lys-63-specific deubiquitinase BRCC36	BRCC3		
O18977_BOVIN	Tenascin-X	TN-X		
A6H7D3_BOVIN	KRT18 protein (Fragment)	KRT18		
J9ZXG5_BOVIN	Integrin alpha V subunit			
B0JYN3_BOVIN	L-lactate dehydrogenase	LDHB		
MB211_BOVIN	Protein mab-21-like 1	MAB21L1		
E1B7R4_BOVIN	Eukaryotic translation initiation factor 3 subunit A	EIF3A	Biological regulation/Metabolic process	Binding/Translation regulator activity
C. List of 46 unique proteins identified in exosomes of ICAR cultured at 8 % O_2_				
Protein ID	Name	Gene Name	Biological Process (Total # Gene 22; Total #Function 49)	Molecular function (Total # Gene 22; Total #Function 28)
F1MMD7_BOVIN	Inter-alpha-trypsin inhibitor heavy chain H4	ITIH4		
F1N3A1_BOVIN	Thrombospondin-1	THBS1		
PLMN_BOVIN	Plasminogen	PLG	Biological regulation/localization/Metabolic process/Response to stimulus	Binding/Catalytic activity/Enzyme regulator activity/Receptor activity
F1MYN5_BOVIN	Fibulin-1	FBLN1	Cellular process/Developmental process	Binding
F1MNV5_BOVIN	Kininogen-1	KNG1		
EF1A1_BOVIN	Elongation factor 1-alpha 1	EEF1A1	Biological regulation/Metabolic process	Binding/Catalytic activity/Translation regulator activity
ITAV_BOVIN	Integrin alpha-V	ITGAV	Biological adhesion	
F1MK44_BOVIN	Integrin alpha-5	ITGA5		
TTHY_BOVIN	Transthyretin	TTR	localization	Transporter activity
F1MC45_BOVIN	Complement factor H (Fragment)	CFH		
J9QD97_BOVIN	Periostin variant 9			
ACTS_BOVIN	Actin, alpha skeletal muscle	ACTA1	Cellular component organization or biogenesis/Cellular process/Developmental process/localization	Structural molecule activity
E1B9K1_BOVIN	Polyubiquitin-C	UBC		
A7YWR0_BOVIN	Apolipoprotein E	APOE		
FA9_BOVIN	Coagulation factor IX	F9	Apoptotic process/Biological regulation/Developmental process/Immune system process/ localization/Metabolic process/Multicellular organismal process/Response to stimulus	Binding/Catalytic activity/Enzyme regulator activity/Receptor activity
COMP_BOVIN	Cartilage oligomeric matrix protein	COMP		
K2C80_BOVIN	Keratin, type II cytoskeletal 80	KRT80	Cellular component organization or biogenesis/Cellular process/Developmental process	Structural molecule activity
TRFE_BOVIN	Serotransferrin	TF	localization/Metabolic process	Catalytic activity
K4JDR8_BOVIN	Alpha-2-macroglobulin variant 5	A2M		
Q32P72_BOVIN	CP protein (Fragment)	CP		
J9ZW47_BOVIN	Integrin beta			
F1MM86_BOVIN	Complement component C6	C6		
E1BI02_BOVIN	Fibromodulin	FMOD		
VNN1_BOVIN	Pantetheinase	VNN1	Biological adhesion/Cellular process/Metabolic process	Catalytic activity
G3X807_BOVIN	Histone H4 (Fragment)		Cellular component organization or biogenesis/Cellular process/Metabolic process	Binding
MOT1_BOVIN	Monocarboxylate transporter 1	SLC16A1	Cellular process/localization	Transporter activity
TF_BOVIN	Tissue factor	F3	Biological regulation/Cellular process/Response to stimulus	Binding/Receptor activity
HS71L_BOVIN	Heat shock 70 kDa protein 1-like	HSPA1L	Metabolic process/Response to stimulus	
Q3ZCA7_BOVIN	Guanine nucleotide binding protein (G protein), alpha inhibiting activity polypeptide 3	GNAI3	Biological regulation/Cellular process/Metabolic process/Response to stimulus	Binding/Catalytic activity
IDHC_BOVIN	Isocitrate dehydrogenase [NADP] cytoplasmic	IDH1		
Q1PBC8_BOVIN	CD14 (Fragment)			
F1MJJ8_BOVIN	Radixin (Fragment)	RDX		
IF4A2_BOVIN	Eukaryotic initiation factor 4A-II	EIF4A2	Biological regulation/Metabolic process	Binding/Catalytic activity/Translation regulator activity
C1QB_BOVIN	Complement C1q subcomponent subunit B	C1QB		
A6QPD4_BOVIN	LOC790886 protein	LOC790886		
CTL2_BOVIN	Choline transporter-like protein 2	SLC44A2	localization	Transporter activity
HPCL1_BOVIN	Hippocalcin-like protein 1	HPCAL1	Cellular process/Multicellularorganismal process	
Q24K07_BOVIN	Vacuolar protein sorting 11 homolog (S. cerevisiae)	VPS11		
Q5H9M6_BOVIN	Dynein heavy chain (Fragment)	Bv2		
Q864S1_BOVIN	Cathepsin C (Fragment)			
Q4ZJS0_BOVIN	MHC class I antigen (Fragment)	BoLA-N		
Q58CZ4_BOVIN	Flotillin 2	FLOT2		
MBL2_BOVIN	Mannose-binding protein C	MBL		Binding
TM214_BOVIN	Transmembrane protein 214	TMEM214		
Q8MIR1_BOVIN	Nicotinic acetylcholine receptor beta 2 subunit (Fragment)	CHRNB2		
Q5E9W1_BOVIN	CDC45-like	CDC45L		

Mass spectrometric (with a set FDR of 5 %) identification of proteins was present in exosomes generated by ICAR cultured at 1 % O_2_ and at 8 % O_2_. Data were subjected to ontology and pathway analysis using PANTHER and gene ontology algorithms and classified based on biological process and molecular function

## Discussion

A successful pregnancy is dependent of having a quality embryo and a receptive uterus synergizing with a synchronized crosstalk between the endometrium and embryo. Any insults or disturbances to its normal course can compromise implantation and the ability for the growing fetus to develop properly in the uterus [26]. The endometrium clearly has important functions in dairy cow pregnancy and we have now shown that exosomal release (30–120 nm) is part of its armamentarium which has analogous properties to similar tissues of other mammalian species.

In the present case, we have shown for the first time the effects of hypoxia on the biological activities of endometrial ICAR cells, including actions on the release and protein content of exosomes. Although it remains to be determined whether exosomes released from ICAR cells at different oxygen tensions also serve different functional goals, our data underscore that the content of exosomes may reflect the physiological state of the cells.

Our non-exosomal characterization of the ICAR cells indicated that the migration and proliferative capacity of ICAR cells decreased, while activation of apoptotic caspase-3 was enhanced at 1 % O_2_ (hypoxia), compared with an oxygen tension that was close to the bovine endometrial physiological oxygen levels (8 % O_2_; [38]). Moreover, the effect on migration was greater when exposed at 1 % O_2_ [39]. Interestingly, no relationship between oxygen tension and cell proliferation and apoptosis was observed in this previous study. Differences in cell types may explain this observation. Ito et al. described the rate of proliferation of human mesenchymal stem cell (MSCs) was observed to be highest in 5 % O_2_ and the lowest in < 0.1 % O_2_ conditions [40]. The MSCs at severely induced hypoxic conditions (<0.1 % O_2_), showed a decrease in proliferative ability, but were able to maintain viability for at least 48 h through increased glucose availability, to facilitate the generation of energy. Similar results were obtained from an airway smooth muscle study [41]. Hence, our cells have relatively normal proliferation responses to decreased oxygen tension.

Our study suggests that exosomes can serve as a vector for signaling molecules that harbor a variety of bioactive molecules including proteins at the conceptus-endometrial interface and that has the potential to modulate the functions of targeted cells during early pregnancy. Endometrial exosome release may also be modulated during an insult such as infection [42, 43]. In the current study we utilized hypoxia (i.e. 1 % O_2_) as a known modulator of exosome release as documented by alteration to both the number of exosomes released as well as differences in the exosomal content (cargo) [24, 27, 29].

In our study, endometrial cells exposed to 1 % O_2_released ~3.6 more exosomes relative to the 8 % O_2_ culture treatment, suggesting that hypoxia modulates cell function, including the release of exosomes. Hypoxia has already been reported to be a stimulus to increase secretion of exosomes by several groups [44–46]. It is also suggested that the protein and RNA content of exosomes can reflect the physiological state of the cell as well as when the cells are in stress condition [47, 48]. However, the initial stress insult that contributed to an alteration of the exosomal content in relation to the functional effects of the subsequent cargo transfer and their role in cell-to-cell communication remains unclear. It is possible that exposure to other stressors such as adverse environmental hazards [49–51] will also increase secretion of exosomes and alter composition of the cargo.

The protein content of exosomes from ICAR cells cultured under the 1 % O_2_ contained unique proteins compared to the contents of the ICAR exosomes cultured at 8 % O_2_. Our proteomic analyses detected the presence of tetraspanin-6 (TSPAN6), disintegrin and metalloproteinase domain-containing protein 10 (ADAM10) that are only unique to exosomes of ICAR cultured at 1 % O_2_. These proteins are involved in the biological processes for reproduction. Interestingly, to evaluate TSPAN6, belonging to the transmembrane 4 superfamily that mediate the regulation of signal transduction events, as well as the disintegrin-like metalloproteinase ADAM10 which participates in ectodomain shedding activity could provide great insights into their functional role and regulation that is important for reproduction.

Studies using immunohistochemistry of human placental explants [52] have demonstrated that ADAM10 expression is significantly increased in preeclamptic placentas compared with normal placentas. Up-regulation of ADAM10 could induce placental release of soluble vascular endothelial growth factor receptor-1 (sFlt-1) and this cascade is associated with endothelial dysfunction, suggesting the significant role of oxidative change in preeclamptic placentas. ADAM10 is also a sheddase [53] that could induce CD46 shedding attributed to cell apoptotic processes [54], as well as mediate E-cadherin shedding affecting cellular adhesion and cell migration [55].

Mass spectrometry detection of pantetheinase (VNN1) in exosomes was unique to ICAR cultured at 8 % O_2_. VNN1 is an enzyme that hydrolyses pantetheine to form pantothenic acid (a precursor of coenzyme A) and the antioxidant cysteamine [56]. VNN1 could promote tissue inflammation through peroxisome proliferator-activated receptor gamma as well as modulate levels of glutathione [57]. It is proposed that VNN1 have innate immune functions and might contribute to tissue injury in endometritis [58, 59]. VNN1 was also reported being involved in proteolysis and can denature proteins by reducing disulfides [60], suggesting that it may have a role in regulating uterine receptivity for implantation and trophoblast invasion [61].

Mass spectrometry detected kininogen-2 (KNG2) in exosomes generated by ICAR cells cultured at either 1 or 8 % O_2_. KNG2 is a precursor protein to high molecular weight kininogen, low molecular weight kininogen and bradykinin and the concentration were reported to fluctuate during ovulation, pregnancy, and parturition [62]. Studies also showed that the release of vasoactive bradykinins from high molecular weight kininogen and low molecular weight kininogen are responsible for micro-vascular permeability and vascular growth, which plays an essential role *in utero*-placental vasculature and angiogenesis, necessary for embryonic and fetal survival [63].

## Conclusion

Our present findings show that ICAR cell function, release of exosomes and exosomal content can be altered when subjected to adverse stimuli. These findings should be expanded to include cells of endometrial epithelial origin, interactions between these cells (i.e. stromal—epithelial crosstalk) and in the presence of common pathophysiological factors associated with reduced fertility (e.g. infectious or inflammatory agents). The identification of unique proteins (by mass spectrometry) in exosomes of ICAR cultured at 1 % O_2_ compared to 8 % O_2_ suggests that the cells respond and release proteins encapsulated within the exosomes to signal the environment in which they live. It is hoped that identification of unique proteins in exosomes following stimulation by factors affecting the physiological condition of cows may lead to novel targets for manipulation to aid fertility. Moreover, investigations into the release, uptake and content of exosomes may offer the opportunity to evaluate maternal-fetal crosstalk.

## Abbreviations

ADAM 10: Metalloproteinase domain-containing protein 10
DTT: Dithiothreitol
FDR: False discovery rate
ICAR: Intercaruncular stromal cell
KNG2: Kininogen-2
LDH: Lactate dehydrogenase
MS/MS: Mass spectrometry/mass spectrometry
PANTHER: Protein analysis through evolutionary relationships
PBS: Phosphate buffered saline
PGF_2α_: Prostaglandin F_2α_
PVDF: Polyvinylidene fluoride
RIPA: Radioimmunoprecipitation assay buffer
RWD: Relative wound density
sFLT-1: Soluble vascular endothelial growth factor receptor-1
TSPAN6: Tetraspanin-6
VNN1: Pantetheinase
